# Combined serum anti-SSA/Ro and salivary TRIM29 reveals promising high diagnostic accuracy in patients with primary Sjögren’s syndrome

**DOI:** 10.1371/journal.pone.0258428

**Published:** 2021-10-08

**Authors:** Maria L. Sembler-Møller, Daniel Belstrøm, Henning Locht, Anne Marie L. Pedersen

**Affiliations:** 1 Faculty of Health and Medical Sciences, Department of Odontology, Section for Oral Biology and Immunopathology, University of Copenhagen, Copenhagen, Denmark; 2 Faculty of Health and Medical Sciences, Department of Odontology, Section for Clinical Oral Microbiology, University of Copenhagen, Copenhagen, Denmark; 3 Department of Rheumatology, Frederiksberg Hospital, Frederiksberg, Denmark; National Institute of Dental and Craniofacial Research, UNITED STATES

## Abstract

**Objectives:**

To determine the diagnostic potential of simultaneous presence of serum anti-SSA/Ro and upregulated salivary protein biomarkers in patients with primary Sjögren’s syndrome (pSS).

**Methods:**

Previous proteomics data on the intensity of neutrophil elastase, calreticulin, tripartite motif containing protein 29 (TRIM29), clusterin and vitronectin provided basis for performing extended analysis. Protein data was obtained by liquid chromatography tandem mass spectrometry technique in whole saliva from 24 patients with pSS and 16 patients having symptoms of pSS, but not fulfilling the American College of Rheumatology/European League against Rheumatism classification criteria (non-pSS). Serum anti-SSA/Ro antibody was measured using enzyme-linked immunosorbent assays. Receiver operating characteristic curve (ROC) value was calculated for combined biomarkers.

**Results:**

Simultaneous presence of serum anti-SSA/Ro and upregulated salivary TRIM29 provided the most optimal combination with an area under curve (AUC) of 0.995 (95% CI 0.98–1.00, *p =* 2.0E-7 and standard error 0.007) and combinations of sensitivity and specificity within the interval of 91–100%. ROC analysis showed that salivary levels of TRIM29 alone enabled differentiation between pSS and non-pSS with an area under curve (AUC) of 0.88 (95%CI 0.77–1.00). All patients with pSS and 3 non-pSS patients were serum anti-SSA/Ro positive.

**Conclusions:**

Simultaneous presence of serum anti-SSA/Ro and upregulated salivary TRIM29 provided a high diagnostic accuracy exceeding that of currently available tools used in pSS diagnostics. This biomarker combination represents a promising less invasive diagnostic tool for pSS. The clinical applicability of TRIM29 needs further testing in independent cohorts using relevant analytical techniques.

## Introduction

Primary Sjögren’s syndrome (pSS) is an autoimmune disease characterized by immune-mediated inflammation directed by the exocrine glands. The diagnostic work-up is complex and time-consuming. Presence of circulating serum antibodies against SSA/Ro is currently one of the best biomarkers for pSS, and found in approx. 70% of the patients [1]. It has been shown that anti-SSA/Ro antibodies may be present in serum up to 18–20 years prior to the diagnosis of pSS [2]. The SSA/Ro antigen includes two distinct components, i.e. SSA/Ro52 (TRIM21) and SSA/Ro60 (TROVE), with SSA/Ro52 being the most common in pSS. Anti-SSA/Ro antibodies may also be present in other inflammatory rheumatic diseases, including systemic lupus erythematosus, rheumatoid arthritis, systemic sclerosis, primary biliary cirrhosis and myositis [3,4]. Accordingly, the sensitivity and specificity of anti-SSA/Ro antibodies have previously been reported to be 83.7 (95%CI 78.0–89.3) and 91.5 (95% CI 87.8–94.9), respectively [5]. TRIM21 is part of the TRIM-protein family, whereas most of the TRIM proteins are induced by interferons, which are signaling molecules considered to play a crucial role in the initial phases of the pathogenesis of pSS [6]. Furthermore, TRIM proteins are involved in recognition of pathogens and regulation of transcriptional pathways in host defense [7]. In a recently published study of ours, we identified a panel of upregulated salivary proteins including neutrophil elastase, calreticulin, TRIM29, clusterin and vitronectin [8]. Among this panel of potential protein biomarker candidates, additional statistical analyses presented in this brief report shows that upregulated TRIM29 seems to represent a remarkably good marker for pSS when combined with presence of anti-SSA/Ro in serum. Although dysregulated TRIM29 has not previously been associated to pSS, it is noteworthy that this particular protein is part of the same protein family as TRIM21 (SSA/Ro52), and may thus share important features and biological roles in the pathogenesis of pSS.

Therefore, we aimed here to evaluate the diagnostic potential of simultaneous presence of serum anti-SSA/Ro antibodies, being the most optimal single biomarker at present, in combination with the previously identified salivary protein biomarker candidates to discriminate between pSS and symptom controls (non-pSS).

## Materials and methods

### Study design and population

The study design and population has previously been described in details [8]. Prior to inclusion, all patients received oral and written information and signed an informed consent. Furthermore, data on salivary protein levels and presence of anti-SSA/Ro have been published previously [8]. This short report therefore presents new results based on additional statistical analyses. In brief, the original study was conducted as a cross-sectional case control study, including a cohort of 40 patients. The patients were included consecutively after being referred from rheumatology or ophthalmology out-patient clinics, private practicing rheumatologists or dentists, when a diagnosis of pSS was suspected. Inclusion criteria comprised an age interval between 18–75 years and presence of symptoms of oral and/or ocular dryness. Exclusion criteria were pregnant and nursing women and presence of secondary Sjögren’s syndrome. The oral clinical examinations, salivary gland biopsy and sample collection were carried out by one examiner (MLSM) with assistance from a senior investigator (AMLP) from October 2016 through December 2017 at the Clinic of Oral Medicine, Department of Odontology at the University of Copenhagen. All samples were collected between 10:00 and 12:00 a.m. to avoid potential influence from diurnal variations in saliva secretion [9].

The comparison of groups were made between patients that fulfilled the American College of Rheumatology/European League against Rheumatism (ACR-EULAR) classification criteria for pSS and patients presenting symptoms and signs of pSS, but who did not fulfill the ACR-EULAR classification criteria (non-pSS) [10]. In total, 24 patients were classified by having pSS and 16 patients were non-pSS. The study was performed in accordance with the Declaration of Helsinki and approved by the Ethical Committees for the Region of Copenhagen, Denmark (H-16035289) and the Danish Data Protection Agency.

### Assessment of serum anti-SSA/Ro antibody

Serum IgG antibodies against SSA/Ro were measured in each patient serum sample using enzyme-linked immunosorbent assays (ELISA) at the clinical biochemical labs in the Copenhagen region at the time of diagnostic work-up for pSS [8]. Values < 7 kU/L were considered negative, 7–10 kU/L were inconclusive and > 10 kU/L were positive.

### Assessment of salivary protein intensities

Chewing-stimulated whole saliva was collected from each patient followed by storage at -80°C until further proteome analysis (for a detailed description of the processing of saliva samples and the proteome analysis [8]). In brief, all saliva samples were analyzed by proteomics using liquid chromatography tandem mass spectrometry. The integrated search engine and a reversed database approach was used with appliance of a 1% false discovery rate at both peptide and protein level. The data was searched against the UniProt human reference proteome database. The mass spectrometry proteomics data have been deposited to the ProteomeXchange Consortium via the PRIDE [11] partner repository with the dataset identifier PXD016231.

### Statistical analysis

Raw-files from mass spectrometry analysis were processed with MaxQuant version 1.5.0.38, and comparison of protein intensities between groups was performed using Perseus software version 1.6.2.3. The diagnostic performance of biomarker candidates and combination of biomarkers with presence of anti-SSA/Ro antibody positivity in serum was conducted using receiver operating characteristic curve (ROC) analysis using SPSS statistics (IBM) software version 26. To calculate the AUC for simultaneous presence of serum anti-SSA/Ro antibodies and salivary levels of the protein biomarker candidates, the values were converted by logistic regression followed by a calculation of the combination ROC of the relevant biomarkers. A *p* value <0.05 was considered statistically significant.

## Results

As previously shown, the two groups did not differ with regard to age, gender, symptoms of oral and ocular dryness or presence of keratoconjunctivitis sicca (determined by means of Schirmer’s test and Lissamin green staining) [8]. The unstimulated and chewing-stimulated whole saliva flow rates were significantly lower in the pSS than in the non-pSS group. Presence of hyposalivation, serum anti-SSA/Ro antibodies and focus score ≥1 in labial salivary gland biopsies were significantly more prominent in the pSS group compared to non-pSS (Table 1) [8]. A detailed overview of the clinical characteristics of each patient is provided in Table 2.

**Table 1:** Demographic and clinical data based on groups [8].

	pSS (n = 24)	non-pSS (n = 16)	*p* value
**Age (years)** §	55±11	53±16	0.573
**Gender (F/M)**	22/2	14/2	1.00
**Xerostomia (yes/no)**	20/4	15/1	0.631
**Ocular dryness (yes/no)**	19/5	12/4	1.00
**Keratoconjunctivitis sicca (yes/no)**	20/4	12/4	0.690
**Hyposalivation (yes/no)**	19/5	7/9	**0.041**
**Anti-SSA positive (yes/no)**	24/0	3/13	**< 0.0001**
**Focus score ≥ 1.0 (yes/no)**	7/17	0/16	**0.029**
**UWS (ml/min)** *	0.04 (0–0.39)	0.15 (0–0.37)	**0.023**
**SWS (ml/min)** *	0.43 (0.07–1.94)	1.03 (0.22–2.34)	**0.038**

§ given as mean ± standard deviation.

* given as median (range). Xerostomia: Symptoms of oral dryness; UWS: Unstimulated whole saliva flow rate; SWS: Stimulated whole saliva flow rate; Hyposalivation: UWS ≤ 0.10 ml/min; bold text: Statistically significant difference.

**Table 2:** Demographic and clinical data based on individuals.

Patient	Age	Gender	UWS	SWS	Focus score	Dry eyes	Dry mouth	KCS	Hypo-salivation	Anti-SSA	ACR/ EULAR
**1**	50	F	0.00*	0.14	1.1	1	1	1	1	1	1
**2**	65	F	0.00*	0.24	0	0	1	1	1	1	1
**3**	46	F	0.00*	0.09	12	1	1	1	1	1	1
**4**	54	F	0.00*	0.24	0	1	0	0	1	1	1
**5**	44	F	0.01	0.08	12	1	1	1	1	1	1
**6**	64	F	0.07	1.11	0	1	1	1	1	1	1
**7**	65	F	0.06	0.82	0	1	1	1	1	1	1
**8**	61	F	0.00*	0.50	1	1	1	1	1	1	1
**9**	60	F	0.24	0.63	0.2	1	0	1	0	1	1
**10**	43	F	0.06	0.38	0	0	1	0	1	1	1
**11**	57	F	0.21	0.13	0	1	1	1	0	1	1
**12**	64	F	0.00*	0.07	1.3	1	1	1	1	1	1
**13**	27	F	0.04	0.46	1.2	1	1	1	1	1	1
**14**	56	F	0.00*	0.13	3.8	1	1	1	1	1	1
**15**	75	F	0.05	1.42	0	1	1	1	1	1	1
**16**	64	F	0.10	1.72	0	0	0	0	1	1	1
**17**	66	M	0.10	1.50	0.8	1	1	0	1	1	1
**18**	55	F	0.04	0.22	0	1	1	1	1	1	1
**19**	58	F	0.39	1.94	0	1	0	1	0	1	1
**20**	49	F	0.01	0.13	0.7	1	1	1	1	1	1
**21**	35	F	0.22	0.86	0.4	1	1	1	0	1	1
**22**	42	M	0.03	0.47	0.3	1	1	1	1	1	1
**23**	61	F	0.00*	0.40	0.9	1	1	1	1	1	1
**24**	63	F	0.36	1.30	0.6	1	1	1	0	1	1
**25**	70	F	0.02	0.22	0	1	1	0	1	0	0
**26**	27	F	0.00*	0.28	0	1	1	1	1	0	0
**27**	63	F	0.31	0.74	0	0	1	1	0	0	0
**28**	51	F	0.13	1.15	0	1	1	1	0	0	0
**29**	47	F	0.37	1.62	0	1	1	0	0	1	0
**30**	72	F	0.24	2.34	0	0	0	0	0	1	0
**31**	45	M	0.08	0.43	0.1	1	1	0	1	0	0
**32**	21	F	0.22	0.69	0	1	1	1	0	0	0
**33**	54	F	0.01	0.29	0	1	1	1	1	0	0
**34**	35	F	0.16	0.97	0.2	1	1	1	0	0	0
**35**	57	F	0.26	1.37	0	1	1	1	0	0	0
**36**	69	F	0.06	0.76	0	1	1	1	1	0	0
**37**	63	F	0.07	1.08	0	1	1	1	1	0	0
**38**	58	F	0.22	1.22	0	1	1	1	0	0	0
**39**	70	M	0.07	1.10	0	1	1	1	1	0	0
**40**	42	F	0.34	1.49	0.5	1	1	0	0	1	0

UWS: Unstimulated whole saliva (ml/min); SWS: Chewing-stimulated whole saliva (ml/min); Dry eyes and mouth items: Reported symptoms; KCS: Keratoconjunctivitis sicca; Hyposalivation: Saliva flow rate ≤ 1.50 ml/15min.

*immeasurable saliva flow.

**Fig 1:**
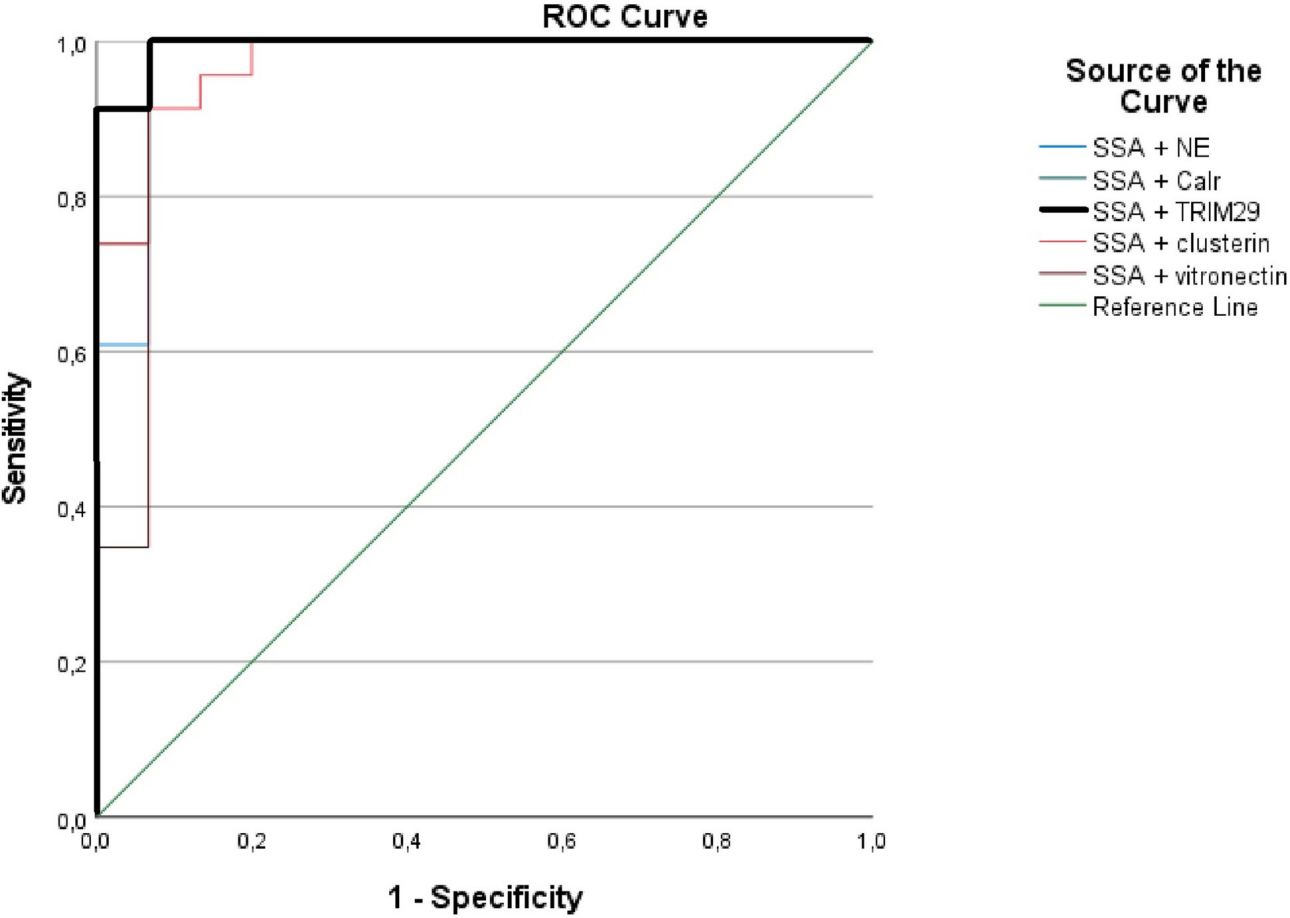
Receiver operating characteristic curve showing the largest area under the curve (upper left corner) of 99.5% for anti-SSA/Ro + TRIM29 combination curve (bold black line).

ROC analysis showed that the combination of upregulated TRIM29 levels in saliva and presence of anti-SSA/Ro antibodies in serum yielded the highest ROC value with an AUC of 0.995 (95%CI 0.98–1.00, *p =* 2.0E-7 and standard error 0.007) (Fig 1). The most optimal sensitivity and specificity for this combination was within the interval of 91–100% (Table 3).

**Table 3:** Coordinates of anti-SSA/TRIM29 combination curve.

**Sensitivity**	100%	100%	100%	93.8%	93.8%	93.8%	87.5%	81.3%
**Specificity**	82.6%	87.0%	91.3%	91.3%	95.7%	100%	100%	100%

This combination showed to be superior to the other protein biomarker candidates, i.e. neutrophil elastase, calreticulin, clusterin and vitronectin (Table 4 and Fig 1).

**Table 4:** Receiver-operating characteristic curve analysis of combined biomarkers.

Biomarkers	AUC	Std. Error	*p* value	95% Confidence Interval	
				Lower Bound	Upper Bound
**Anti-SSA/Ro + neutrophil elastase**	.974	.027	1.0E-6	.921	1.000
**Anti-SSA/Ro + calreticulin**	.983	.019	6.6E-7	.946	1.000
**Anti-SSA/Ro + TRIM29**	.995	.007	2.0E-7	.980	1.000
**Anti-SSA/Ro + clusterin**	.974	.022	1.0E-6	.932	1.000
**Anti-SSA/Ro + vitronectin**	.957	.043	3.0E-6	.872	1.000

**Fig 2:**
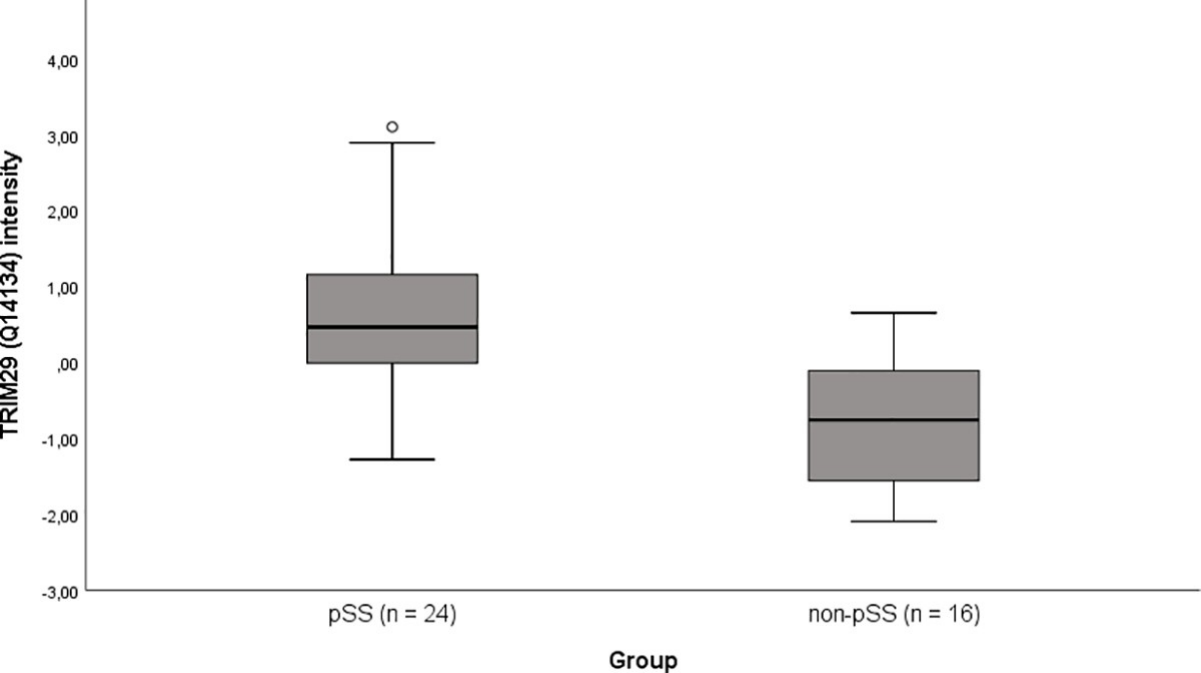
Box plot of salivary TRIM29 in pSS and non-pSS. Box plot illustrating the salivary TRIM29 intensity in the two groups after subtracting the median value across samples from individual samples. The group difference was 1.54, indicated by mean intensity of pSS–non-pSS. Mean intensity in pSS = 0.73 and non-pSS = -0.81.

**Fig 3:**
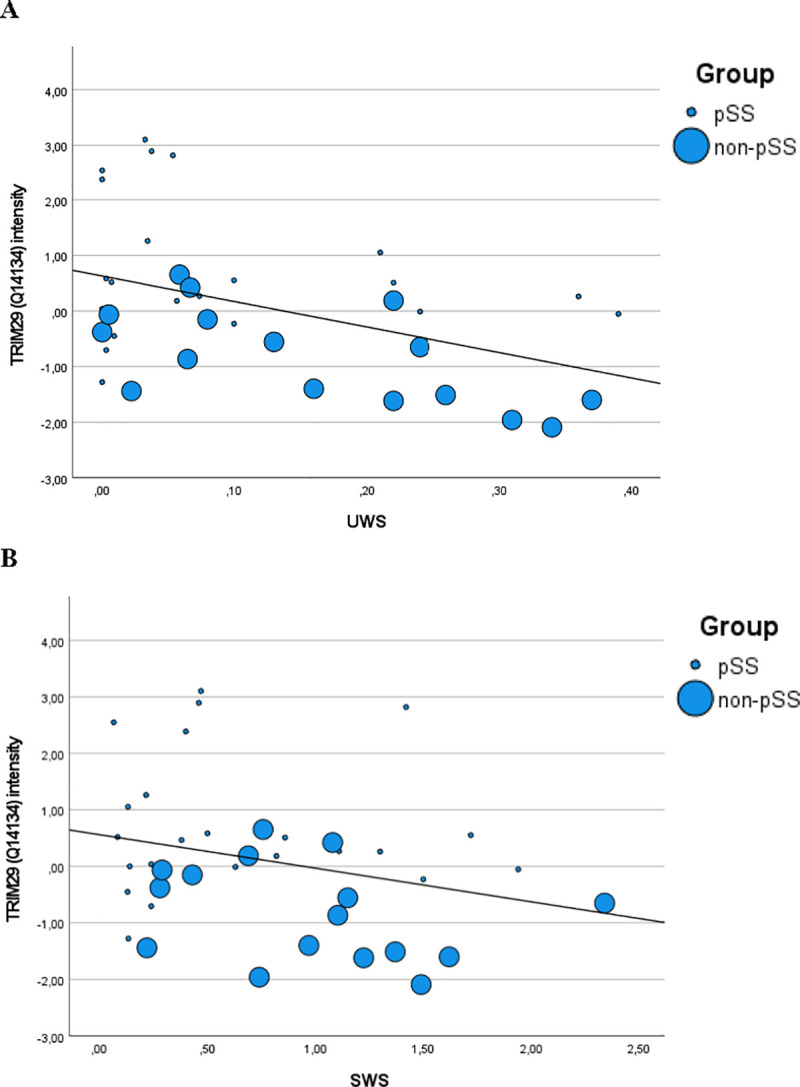
Correlation of salivary TRIM29 and saliva flow rates. Scatter plot illustrating the relation between salivary TRIM29 intensity and (A) unstimulated saliva flow rate (UWS) as well as (B) stimulated saliva flow rate (SWS). Small dots represent pSS patients and larger dots represent non-pSS patients. A weak, but statistically significant correlation (*p* = 0.024) was observed between TRIM29 and UWS, but not between TRIM29 and SWS.

The levels of TRIM29 (UniProt ID Q14134) was significantly higher in the pSS group than in the non-pSS group (*p* = 0.0001 before and p = 0.019 after Benjamini-Hochberg correction for multiple dependent analysis) (Fig 2). TRIM29 alone yielded an AUC of 0.88 (95%CI 0.77–0.995, *p =* 0.0001 and standard error 0.06) in discriminative performance between pSS and non-pSS [8]. The salivary level of TRIM29 showed a weak correlation with unstimulated whole saliva flow rate (R = -0.36, *p* = 0.024) and no correlation with stimulated whole saliva flow rate (Fig 3). Based on this cohort, the presence of anti-SSA/Ro antibodies in serum showed an AUC of 0.906 with a sensitivity of 100% and a specificity of 81%.

## Discussion

The current diagnostic work-up of pSS is time-consuming and requires invasive and costly procedures that involve a multidisciplinary collaboration between several specialists. Therefore, less invasive and simpler ways to diagnose and classify patients with pSS are warranted.

Presence of anti-SSA/Ro autoantibodies in serum and/or a focus score equal to or above one, representing focal lymphocytic inflammation of the minor labial salivary glands, are currently the most important diagnostic tools for the diagnosis of pSS [10]. There is an increasing interest in identifying less demanding and invasive diagnostic biomarkers, which may replace the necessity of an invasive procedure like a labial salivary gland biopsy.

Whole saliva is a mixed fluid comprising secretory products from the main affected organs in pSS, e.g. the salivary glands. Thus, it is likely that saliva reflects essential immunological changes, including an altered protein expression within the glands, which potentially can be used as a non-invasive and early diagnostic biomarker for pSS [12].

The biological function and clinical significance of upregulated TRIM29 in pSS are unclear. Findings of a recent and very comprehensive study suggest that TRIM29 plays a role in DNA virus infection, particularly in Epstein-Barr virus (EBV) infection [13]. Thus, it was shown that EBV, in human airway epithelial cells, induces TRIM29 to inhibit innate immune activation, and that knockout of TRIM29 (in mice) resulted in an enhanced type I interferon production, leading to EBV clearance. In addition, lung tissue from TRIM29-knockout mice revealed less inflammation than that from wild-type (TRIM29 positive) mice following adenovirus infection. The authors suggest that TRIM29 acts as a negative regulator of DNA-sensing, which most likely play an important role in EBV-induced cancers and autoimmune diseases [13]. EBV has previously been suggested as an environmental trigger of pSS, and DNA from EBV has been observed in salivary gland biopsies from patients with pSS more frequently than in those from healthy controls, indicating that EBV activation may drive the inflammation and immune response in pSS [14,15]. Consequently, TRIM29 most likely plays a role in the initiation and maintenance of pathophysiological processes in pSS. This short communication is based on a post hoc statistical analysis. The finding of this study displays promising perspectives, as the identification of a single upregulated protein in saliva (TRIM29) in combination with the presence of the already validated serum biomarker, anti-SSA/Ro antibody, demonstrates potential not only to simplify the diagnostic work-up, but also to increase the diagnostic accuracy of pSS significantly. This combination enabled 99.5% accurate discrimination of patients with pSS and non-pSS. Furthermore, a significant correlation was found between salivary TRIM29 intensity and UWS flow rates, but not between TRIM29 and SWS flow rates. However, it seems unlikely that the upregulation of TRIM29 is merely related to lower salivary flow rates. A large variety of systemic diseases and medical conditions, and also intake of certain medications and/or polypharmacy, and cancer therapy, particularly radiotherapy to the head and neck region are associated with reduced UWS flow rates [16]. Moreover, in our study, approx. 78% of the non-pSS patients had hyposalivation (Tables 1 and 2).

Our findings are based on a small study population, and further validation in larger and independent cohorts and multicenter studies is needed. In addition, the protein data in this brief report is based on a comprehensive proteomics technique that is currently costly and time consuming. Future use for diagnostics and screening purposes therefore requires development of easy applicable point-of-care test kits, such as ELISA, to identify upregulated TRIM29. Current work is now focused on evaluation and optimization of e.g. ELISA kits, that can facilitates the use of saliva for protein analyses, including salivary TRIM29 as a potential biomarker in the diagnosis of pSS.

The limitations of this study are the small study population and also the lack of information regarding the specific Ro-subtype, i.e. Ro60 and Ro52. Therefore, in an upcoming study, including validation of the potential of salivary TRIM29 intensity in combination with anti-SSA/Ro antibodies, the Ro antibodies will be fractionated to determine if this particular association is tied up on presence of Ro60/TROVE or Ro52/TRIM21 exclusively.

In addition, increasing evidence supports a local production and presence of anti-SSA/Ro antibodies in salivary glands and secretion into saliva. However, the technology applied for detection of these salivary antibodies still needs further evaluation and validation [17]. Nevertheless, it may be speculated that the upregulation of anti-SSA/Ro antibodies in saliva combined with upregulated TRIM29 in saliva could serve as an interesting early-phase and non-invasive combination of salivary biomarkers for pSS in the future.

## Conclusion

In patients with pSS, the simultaneous presence of anti-SSA/Ro antibodies in serum and upregulated TRIM29 in saliva enabled 99.5% differentiation from non-pSS patients with pSS-like symptoms and signs, thereby providing a diagnostic accuracy that may exceed the currently available biomarkers for pSS. However, this is based on a small cohort and costly in-depth proteomic analysis, and the applicability of TRIM29 as a biomarker in pSS diagnostics therefore needs further validation and ideally development of point-of-care tests easy applicable for clinical usage.
